# Challenges in Transitioning from Controlled to Assisted Ventilation in Acute Respiratory Distress Syndrome (ARDS) Management

**DOI:** 10.3390/jcm13237333

**Published:** 2024-12-02

**Authors:** Denise Battaglini, Patricia R. M. Rocco

**Affiliations:** 1 Department of Surgical Sciences and Integrated Diagnostics (DISC), University of Genova, 16132 Genova, Italy; 2 Anesthesia and Intensive Care, IRCCS Ospedale Policlinico San Martino, 16132 Genova, Italy; 3 Laboratory of Pulmonary Investigation, Carlos Chagas Filho Institute of Biophysics, Centro de Ciências da Saúde, Federal University of Rio de Janeiro, Avenida Carlos Chagas Filho, 373, Bloco G-014, Ilha Do Fundão, Rio de Janeiro 21941-598, RJ, Brazil; prmrocco@gmail.com

**Keywords:** ARDS, weaning, mechanical ventilation, transitioning, assisted ventilation, pressure support ventilation, controlled ventilation

## Abstract

Acute respiratory distress syndrome (ARDS) presents significant challenges in critical care, primarily due to its inflammatory nature, which leads to impaired gas exchange and respiratory mechanics. While mechanical ventilation (MV) is essential for patient support, the transition from controlled to assisted ventilation is complex and may be associated with intensive care unit-acquired weakness, ventilator-induced diaphragmatic dysfunction and patient self-inflicted lung injury. This paper explores the multifaceted challenges encountered during this transition, with a focus on respiratory effort, sedation management, and monitoring techniques, and investigates innovative approaches to enhance patient outcomes. The key strategies include optimizing sedation protocols, employing advanced monitoring methods like esophageal pressure measurements, and implementing partial neuromuscular blockade to prevent excessive respiratory effort. We also emphasize the importance of personalized treatment plans and the integration of artificial intelligence to facilitate timely transitions. By highlighting early rehabilitation techniques, continuously assessing the respiratory drive, and fostering collaboration among multidisciplinary teams, clinicians can improve the transition from controlled to assisted MV, ultimately enhancing recovery and long-term respiratory health in patients with ARDS.

## 1. Introduction

Acute respiratory distress syndrome (ARDS) is a severe condition characterized by widespread inflammation in the lungs, leading to significant impairment in gas exchange and respiratory function [1]. While mechanical ventilation (MV) is essential for supporting oxygenation and carbon dioxide removal in ARDS patients [2,3], the transition from controlled to assisted ventilation is complex and may be associated with intensive care unit-acquired weakness, ventilator-induced diaphragmatic dysfunction and patient self-inflicted lung injury [4,5].

The process of transitioning from controlled to assisted ventilation is critical in ARDS management, often consuming up to 40% of a patient’s ICU stay [6]. Successfully shifting to assisted ventilation may re-establish spontaneous breathing, improve oxygenation, reduce muscle atrophy, and minimize the risks associated with prolonged controlled MV [7,8,9]. However, this process is challenging due to the complex physiology of ARDS, which increases the risk of respiratory fatigue and distress [10].

Optimizing this transition requires careful consideration of factors such as respiratory effort, sedation, the arterial partial pressure of oxygen to fraction of inspired oxygen ratio (PaO_2_/FiO_2_) and selection of the appropriate ventilation mode [11].

In this paper, we explore the key challenges in transitioning to assisted ventilation in ARDS and examine the underlying mechanisms, offering practical strategies to support clinicians in enhancing patient outcomes, as well as innovative approaches.

## 2. Lung and Diaphragm Injury During the Transition from Controlled to Assisted Mechanical Ventilation

Several mechanisms can be responsible of patient self-inflicted lung injury (P-SILI) [12]. This mechanism is mediated by well-established physiological processes and can be exacerbated during the transition from controlled to assisted MV. The contributing factors include the following. (1) Excessive global lung stress: increased tidal volume (VT) and transpulmonary pressure (PL) can lead to lung injury. (2) Excessive regional lung stress: in already injured lungs, excessive stress and strain are not uniformly distributed during inflation. This can result in large PL swings in consolidated regions, causing air movement from nondependent to dependent lung regions, a phenomenon known as *pendelluft* [13]. (3) Increased transvascular pressure: PL is negative during spontaneous breathing, which can increase the transvascular pressure, raising the total lung water and inducing pulmonary edema. (4) Patient–ventilator asynchronies: particularly, issues like double triggering and reverse triggering can increase the VT and PL, potentially resulting in *pendelluft* and further lung injury [14,15].

In addition to lung injury, myotrauma to the diaphragm is also a concern during this transition. The mechanisms of diaphragm injury include the following. (1) Excessive unloading: this usually occurs due to excessive over-assistance, leading to suppression of the respiratory drive and disuse atrophy. (2) Excessive concentric loading: higher inspiratory patient effort, dyssynchronies, and inadequate support are common in assisted MV. Vigorous concentric contractions can induce significant muscular stress, leading to muscle inflammation, proteolysis, myofibrillar injury, and sarcolemma disruption. (3) Excessive eccentric loading: eccentric contractions, which occur when a muscle generates contractile tension while lengthening rather than shortening, are substantially more damaging than concentric contractions [16]. In cases of low positive end-expiratory pressure (PEEP) and excessive drops in the end-expiratory lung volume, the diaphragm may contract while lengthening during the expiratory (“post-inspiratory”) phase to avoid atelectasis, a phenomenon known as “expiratory braking” [17]. Specific types of dyssynchrony (e.g., reverse triggering, short cycling, ineffective effort) may elicit eccentric contractions because the diaphragm is activated during the expiratory phase. (4) Excessive PEEP: when the PEEP is excessively high, the sarcomeres may shorten, leading to longitudinal atrophy [18]. Diaphragm ultrasound is a valuable tool for identifying diaphragm changes during the transition between controlled and assisted mechanical ventilation. The mode of ventilation affects diaphragm contraction and thickness [19]. Using a 7.5–10 MHz linear probe positioned at the zone of apposition, parallel to an intercostal space between the 8th and 10th ribs, clinicians can easily measure the diaphragm thickness and thickening fraction. The thickening fraction is calculated in M-mode as follows: (end-inspiratory thickening − end-expiratory thickening)/end-expiratory thickening × 100 [20]. If the diaphragm thickening exceeds 10% during assisted ventilation, an alternative ventilatory strategy with adjusted levels of assistance may be considered. This approach aims to achieve a safe, rapid, shallow breathing index and pressure muscle index, as demonstrated by Bellani and colleagues [19].

## 3. Sedation and Analgesia During the Transition from Controlled to Assisted MV

During the first 48 h of MV, patients with ARDS often exhibit spontaneous breathing, which can facilitate earlier liberation from both MV and the ICU [21]. Sedation is critical to managing this transition. Studies such as the WEAN-SAFE found that higher sedation levels are associated with delayed weaning initiation, which is closely linked to weaning failure [22].

The choice and timing of sedative transitions—such as switching from benzodiazepines to propofol—are crucial for managing the respiratory drive and maintaining patient stability. For instance, propofol has been shown to decrease the VT by 36% without affecting the respiratory rate during unassisted breathing, while benzodiazepines reduce the VT by 28%. In contrast, opioids generally lower the respiratory rate, impacting both the MV and unassisted breathing phases. These findings highlight the importance of selecting sedatives that align with the patient’s current respiratory support needs and overall stability, as each sedative affects respiratory parameters differently and may pose challenges if not adjusted properly during the transition [23].

Patients undergoing prolonged deep sedation while on controlled MV are at a higher risk of failing the transition to assisted breathing. Balzani et al. analyzed 4171 breaths from 48 patients and found that the number of days of deep sedation was independently associated with the composite outcome of either transitioning from light to deep sedation (RASS from 0/−3 to −4/−5) or returning to controlled ventilation within 48 h of spontaneous assisted breathing (aHR 1.15 [1.07–1.24], *p* = 0.0002). The study also highlighted that a COVID-19 ARDS diagnosis significantly contributed to these outcomes (aHR 6.96 [1–48.5], *p* = 0.05) [24].

In patients ventilating in assisted modes, increasing the sedation depth with propofol did not result in significant changes in respiratory timing; however, it did lead to a progressive decrease in the neural drive (observed in both pressure support ventilation (PSV) and neurally adjusted ventilatory assist (NAVA)) and effort (notably in PSV) [25]. Additionally, the use of fentanyl may exacerbate expiratory effort in ARDS patients. Plens et al. demonstrated that higher fentanyl doses were associated with increases in the end-expiratory lung volume (EELV). The rise in the EELV correlated positively with the fentanyl dosage and negatively with changes in the end-expiratory pleural pressures, suggesting active expiratory effort in these patients [26].

Further supporting these findings, Perez et al. reported that in patients with acute respiratory failure of various etiologies, the initial PSV attempt failed 43.7% of the time, with a greater prevalence observed in those with COVID-19. The independent risk factors for first transition failure included the fentanyl dose, COVID-19 diagnosis, prior use of neuromuscular blockers, acidosis, and hypoxemia before spontaneous awakening trials. Interestingly, a higher body mass index emerged as a protective factor, influencing clinical outcomes negatively [27].

Dexmedetomidine effectively reduced agitation. Within six hours of initiating dexmedetomidine treatment, the Motor Activity Assessment Scale (MAAS) scores improved to indicate mild agitation or calm states, with the target MAAS levels sustained at 12 h at most time points. This approach led to successful weaning and extubation in more than half of the patients, and in 75% of episodes, excluding those complicated by clinical deterioration [28]. In a single-center randomized controlled trial (RCT), sedation strategies were compared: switching from midazolam to dexmedetomidine, switching from midazolam to propofol, or continuing midazolam alone. The group transitioned from midazolam to dexmedetomidine demonstrated earlier recovery, faster extubation, lower incidence of delirium, and shorter weaning times compared to the other groups. Additionally, the midazolam-to-propofol group showed shorter recovery, extubation, and weaning times than the group maintained on midazolam [29].

Volatile anesthetics reduce the VT while concurrently increasing the respiratory rate in a dose-dependent manner, with the potential to relieve lung stress and strain in spontaneously breathing patients [30]. They also significantly decrease minute ventilation only at dosages around and above 1 minimal alveolar concentration (MAC), which is greater than the estimated dose of 0.5 MAC required for intensive care sedation [31,32,33]. Moreover, it seems that sevoflurane and alfentanil synergistically reduce minute ventilation [33]. In contrast, volatile anesthetics preserve the respiratory drive better than intravenous sedatives. The Sedaconda trial [32], which compared isoflurane with propofol sedation in critically ill patients, found that 50% of patients sedated with isoflurane were spontaneously breathing on day one compared to 37% with propofol sedation (odds ratio: 1.7 (95% CI: 1.1, 2.6), *p* = 0.013). A subgroup analysis of this trial demonstrated that isoflurane increased the probability of assisted spontaneous breathing by twofold when compared to propofol (risk ratio: 2.4 (95% CI: 1.5–3.7), *p* < 0.001) [34]. While volatile anesthetics increase the ventilatory dead space, the effect is minimal and comparable to ventilation with heat and moisture exchangers. However, this should be considered as a potential limitation of their use.

Relying solely on clinical sedation scores, such as the Richmond Agitation–Sedation Scale (RASS), is insufficient for accurately estimating the respiratory drive [35]. The implementation of quantitative electroencephalogram (qEEG) monitoring may offer predictive value for weaning failure in MV patients [36]. Therefore, current ICU guidelines recommend the use of processed EEG for monitoring sedation, especially in patients undergoing MV and weaning [37].

## 4. Monitoring Effort and Drive During the Transition from Controlled to Assisted Mechanical Ventilation

Monitoring the patient’s respiratory effort is a key step in achieving an effective transition from controlled to assisted MV. Esophageal pressure (Pes) monitoring has become a standard practice for evaluating the effort and work of breathing in ARDS patients undergoing passive MV. The Pes serves as a reliable surrogate for the pleural pressure (Ppl), despite regional variations. The distinct characteristics of assisted and controlled MV result in different pressures: during passive breathing, the PL is typically lower than the airway pressure (Paw) due to positive pleural pressure swings. Conversely, during assisted breathing, the PL can exceed the Paw significantly; this occurs when strong inspiratory efforts create negative swings in the Ppl, leading to an increase in the PL. Consequently, PL measurements may underrepresent lung stress in dependent lung areas when accounting for regional ventilation variability and *pendelluft* phenomena. The *pendelluft* effect can be identified through dynamic swings in the PL (ΔPL), with a ΔPL of 3–8 cmH_2_O considered a critical threshold for mechanical stress in dorsal lung regions under dynamic conditions [38]. To quantify the inspiratory effort, the Pes helps calculate the inspiratory muscle pressure (Pmus) as follows:

Pmus = Pcw − Pes

where Pcw represents the additional pressure needed to overcome the elastic recoil of the chest wall. While the optimal Pmus levels for assisted mechanical breathing remain uncertain, values ranging from 5 to 10 cmH_2_O may prevent diaphragm atrophy [39]. The pressure–time product is a gold standard for measuring respiratory effort, defined as the integral of the Pmus over the duration of inspiration. A pressure–time product cut-off of 50–100 cmH_2_O/s/min reflects acceptable oxygen consumption and effort [40].

Overall, when the level of assistance is altered, critically ill patients react by adjusting the VT, respiratory drive, effort, and respiratory rate. In the past, the respiratory rate was considered a key variable for determining respiratory muscle unloading in response to increasing levels of assistance [41,42]. However, recent findings suggest that while the respiratory rate changes in the same direction as effort, the magnitude of the change and the final respiratory rate do not correlate with the level of effort. This indicates that the respiratory rate plays a limited role in titrating assistance to achieve a target level of effort [43,44].

**Figure 1:**
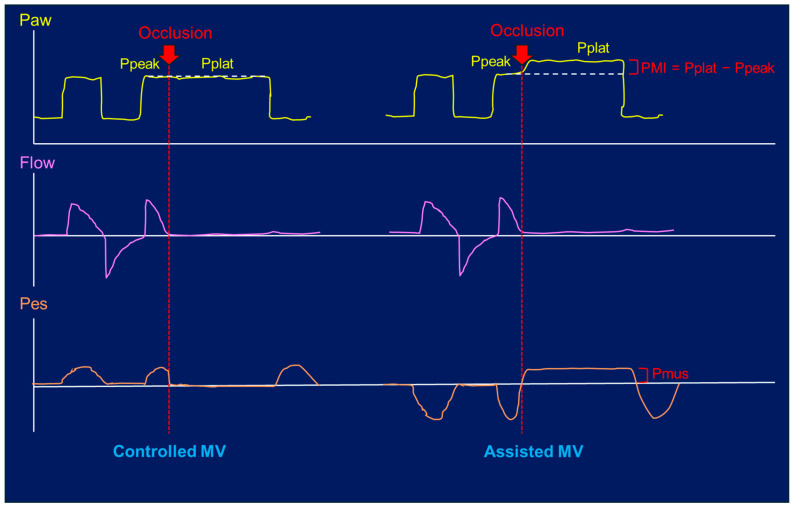
Inspiratory occlusion maneuver in controlled and assisted mechanical ventilation (MV). PMI = pressure muscle index, representing the difference between the Pplat = Plateau pressure and the expected Ppeak = Peak pressure (Pressure support + positive end-expiratory pressure (PEEP)). Pmus: inspiratory muscle pressure; Paw: airway pressure; Pes: esophageal pressure.

Other reliable and less invasive bedside techniques include the inspiratory occlusion maneuver. In passive conditions, this maneuver allows measurement of the plateau pressure (Pplat), which reflects the dynamic lung stress and risk of lung injury. In assisted MV, when a patient exerts significant inspiratory effort, airflow cessation leads to an increase in the Pplat above the Ppeak during the inspiratory hold, indicating an increased esophageal swing. Conversely, if the inspiratory effort is minimal, the small difference between the Ppeak and Pplat suggests that the respiratory muscles exert little effort during the breath. This technique may underestimate the impact of *pendelluft* on P-SILI [45], as in Figure 1.

The expiratory occlusion maneuver in assisted MV can provide measurements of the maximal inspiratory pressure. The swings in the Ppl caused by patient respiratory effort can be evaluated using the airway pressure variations during occlusion (ΔPocc). As long as the patient’s respiratory drive remains unchanged during a transient end-expiratory occlusion, the ΔPocc can be utilized to predict changes in the ΔPes, Pmus, and ΔPL throughout the respiratory cycle [46].

Given the association between high airway occlusion pressure, dyspnea, and increased mortality in critically ill MV patients [47], monitoring the respiratory drive is crucial. The P0.1 measurement, which reflects the airway pressure generated in the first 100 milliseconds of inspiration against an expiratory occlusion, provides insight into the patient’s respiratory drive. P0.1 values below 1.5 cmH_2_O may indicate insufficient respiratory effort, while values above 3.5 cmH_2_O suggest excessive respiratory drive [48]. A recent study demonstrated that both the Pocc and P0.1 are associated with changes in the transpulmonary pressure (R2 = 0.62 and 0.51, respectively) and transdiaphragmatic pressure (R2 = 0.53 and 0.22, respectively). The diagnostic performance of the Pocc and P0.1 in detecting extremes in these parameters ranges from average to outstanding. The Pocc is more accurate at recognizing strong diaphragm effort [49].

A secondary analysis of a prospective observational study using multivariable linear regression identified dyspnea (*p* = 0.037), the respiratory rate (*p* < 0.001), and PaO_2_ (*p* = 0.008) as independent covariates associated with the P0.1. The ninety-day mortality was 33% in patients with P0.1 > 3.5 cmH_2_O, compared to 19% in those with P0.1 between 1.5 and 3.5 cmH_2_O and 17% in those with P0.1 < 1.5 cmH_2_O (*p* = 0.046). After adjusting for major risk factors, the P0.1 was independently associated with 90-day mortality (hazard ratio per 1 cmH_2_O, 1.19 (95% CI, 1.04–1.37); *p* = 0.011). Furthermore, the P0.1 was linked to a longer duration of mechanical ventilation (hazard ratio per 1 cmH_2_O, 1.10 (95% CI, 1.02–1.19); *p* = 0.016). This study suggests a potential association between high respiratory drive and an increased mortality risk [50].

Emerging methods for monitoring spontaneous breathing include assessing diaphragm activity through the electrical activity of the diaphragm (EAdi) in NAVA or noninvasive ultrasound techniques. The EAdi correlates with changes in diaphragm thickness throughout the respiratory cycle, with diaphragm weakness detectable when the thickening fraction (TFdi) falls below 30% during maximal inspiratory effort [51]. A randomized controlled trial (RCT) by Diniz-Silva et al. found that maintaining the VT within protective levels was feasible for 75% of ARDS patients using both NAVA and PSV, although all the patients required continuous sedation, and both modes demonstrated comparable outcomes regarding the VT, respiratory rate, and Paw [52].

Recent therapeutic approaches have introduced the use of neuromuscular blocking agents (NMBAs) for partial paralysis of respiratory muscles while allowing some spontaneous breathing—referred to as partial neuromuscular blockade. This strategy aims to reduce the work of breathing and improve the patient–ventilator synchrony, ultimately minimizing the discomfort and anxiety during the transition phase. Though still in the “proof of concept” stage, evidence suggests that partial neuromuscular blockade may help prevent respiratory muscle fatigue in patients with high respiratory drive. Doorduin et al. demonstrated that during partial ventilatory support, this approach preserved diaphragm function while facilitating lung-protective MV, leading to significant decreases in the VT, transpulmonary pressure, and diaphragm electrical activity, alongside increases in the heart rate, mean arterial pressure, and pH during titration [53]. However, a novel prospective study demonstrated that the depth of the neuromuscular blockade was not associated with the chest wall elastance, inspiratory Pes, or expiratory Pes. This suggests the need for larger studies to confirm the utility of partial neuromuscular blockade in facilitating lung- and diaphragm-protective MV [54]. In a physiological trial investigating the transition from controlled to assisted ventilation, partial NMBAs were administered. Only 6 out of 30 patients met the criteria for lung- and diaphragm-protective ventilation. However, lung- and diaphragm-protective ventilation was achieved in 20 out of 30 patients through a combination of ventilation titration, sedation, sweep gas flow for those on veno-venous extracorporeal membrane oxygenation, and PEEP levels optimized to improve compliance. The use of partial NMBA in patients with refractory excessive effort was well tolerated and effectively met protective ventilation goals [55].

**Figure 2:**
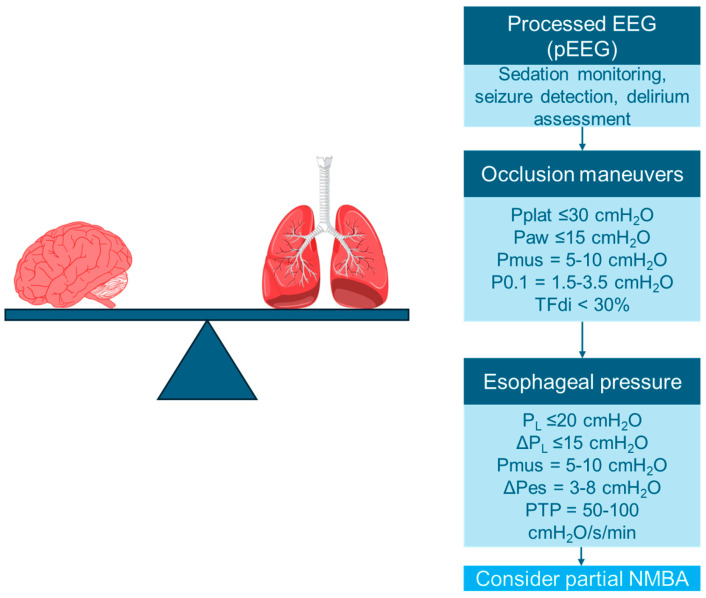
Key strategies for monitoring sedation, drive, and effort during the transition from controlled to assisted mechanical ventilation. Pplat = plateau pressure; Paw = airway pressure; Pmus = muscular pressure; P_L_ = transpulmonary pressure; ΔP_L_ = dynamic transpulmonary pressure; ΔPes = swings in esophageal pressure; PTP = pressure-time product; NMBA = neuromuscular blocking agent. Modified from [48].

The key strategies for monitoring sedation, drive, and effort during the transition from controlled to assisted MV are essential to optimize patient outcomes. By implementing advanced monitoring techniques and therapeutic approaches, clinicians can enhance patient safety and improve the effectiveness of ventilation strategies during this critical phase (Figure 2).

## 5. Innovative Approaches

In ARDS management, innovative approaches to facilitate successful transitions from controlled to assisted ventilation focus on enhancing diaphragm conditioning, personalizing sedation, and integrating advanced monitoring and artificial intelligence (AI)-driven support [56].

To reduce diaphragm atrophy and strengthen respiratory muscles, early rehabilitation with intermittent spontaneous breathing trials (SBTs) during controlled ventilation is recommended [57]. Complementary to SBTs, neuromuscular electrical stimulation has shown potential for strengthening respiratory muscles pre-transition, providing a non-invasive means to preserve muscle function in prolonged controlled ventilation cases [53,58].

Partial neuromuscular blockade may be a promising approach to modulate the respiratory drive and prevent excessive effort, reducing the risk of P-SILI. Paired with advanced monitoring of drive indicators such as the esophageal pressure and airway occlusion pressure, this technique could enable safer and more personalized transitions to assisted ventilation [53].

Advanced monitoring technologies like processed EEG can help fine-tune sedation levels, striking an optimal balance between sedation and spontaneous breathing. This individualized sedation strategy supports smoother, more responsive transitions. In addition, NAVA can synchronize ventilator support with the patient’s neural respiratory drive, providing lung-protective volumes that dynamically adapt to the patient’s needs. This method, combined with continuous monitoring of respiratory mechanics, helps ensure that spontaneous breaths stay within protective thresholds [59,60,61]. Closed-loop sedation and closed-loop MV strategies will probably be able to be integrated in the near future [59,62,63].

Biomarker-driven approaches that can identify patients with elevated inflammatory loads may enable targeted anti-inflammatory therapies (e.g., novel immunomodulators) before transitioning to assisted ventilation. By reducing inflammation, these interventions could decrease the excessive respiratory drive and facilitate a more stable shift to assisted modes [64]. Cornejo et al. found that interleukin (IL)-8, IL-18, and caspase-1 were significantly associated with the frequency of high-magnitude *pendelluft*. These findings suggest that the frequency of high-magnitude *pendelluft* may influence the inflammatory response to inspiratory efforts in ARDS patients transitioning to partial support ventilation [65]. In 2012, Dessap et al. [66] published a trial comparing two different approaches to weaning: one guided by the B-natriuretic peptide (BNP) levels and use of diuretics, and the other following a standard approach for transitioning and fluid management. The BNP-guided group was weaned more quickly, with more ventilator-free days and fewer complications, including a lower need to return from assisted to controlled ventilation (28% vs. 43%) compared to the traditional group. Predictive biomarkers, such as the BNP, may play a role in transitioning and weaning from MV, as the BNP is an easily accessible marker for bedside assessment. Further testing of additional biomarkers in clinical trials is needed to better understand their role in challenging situations, such as transitioning from controlled to assisted ventilation.

AI-driven predictive models that incorporate patient-specific data—such as respiratory mechanics, inflammatory markers, and neural drive indicators—can aid clinicians in determining the optimal timing for transition. These models may lower the risk of failed weaning attempts and provide real-time guidance on ventilatory and sedation adjustments [62,67]. A recent systematic review provided an overview of empirically investigated predictors for weaning failure developing an evidence map. The review included 140 studies investigating 145 predictors, which were categorized into imaging procedures (*n* = 22), physiological parameters (*n* = 61), scores and indices (*n* = 53), and machine learning models (*n* = 9). The rapid shallow breathing index, diaphragm thickening fraction, respiratory rate, PaO_2_/FiO_2_ ratio, and diaphragm excursion were the most frequently investigated predictors. Although these predictors were frequently mentioned, they were often criticized as being poorly researched, with the lack of an international definition for weaning identified as a major limitation [68].

Machine learning techniques may offer a promising solution for predictive approaches by combining computer science and respiratory physiology. Many studies have used machine learning to develop weaning prediction models, but very few specifically in the transition phase between controlled and assisted MV. Hsieh et al. [69] developed a neural network model based on data from 3602 MV patients, achieving an area under the receiver operating curve (AUROC) of 0.85. Furthermore, the same authors created models for simple, prolonged, and difficult weaning, which achieved AUROCs of 0.910, 0.849, and 0.942, respectively [70]. In a data-mining process using AI on prospectively collected data, Manguy et al. found that heart rate variability played a prominent role, in addition to the respiratory drive and body mass index, in determining ventilator weaning outcomes [71]. A novel AI model developed by Liu et al. predicted the success and timing of MV weaning, from intubation to the change in ventilatory mode, and from assist control to support mode. The model identified 11 time frames and predicted weaning success for the nearest time frame. This model was able to reduce the MV duration by 21 h and the ICU stay by 0.5 days, compared to previous studies. However, the weaning success rate was similar to previous findings, highlighting the need for further refinement of weaning models using AI [72].

**Figure 3:**
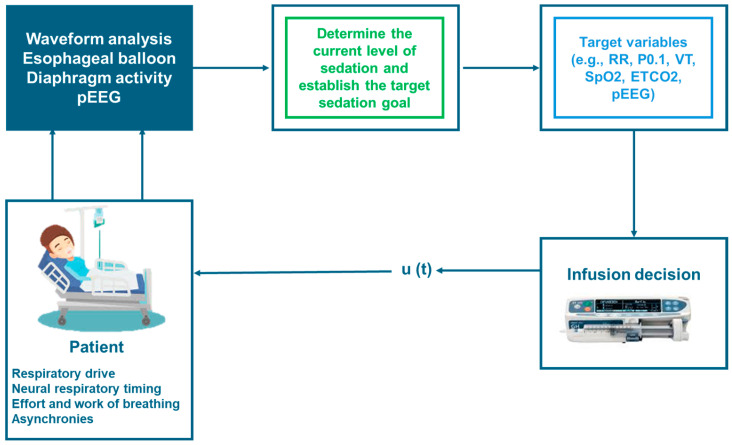
Implementation of closed-loop systems using artificial intelligence for transitioning between controlled and assisted MV. RR = respiratory rate; VT = tidal volume; SpO2 = peripheral saturation of oxygen; ETCO2 = end-tidal carbon dioxide; pEEG = processed electroencephalogram; u(t) = control feedback.

The implementation of closed-loop ventilation modes, which control one or more output variables of the mechanical ventilator, holds promise for enhancing AI and machine learning models in the transition from controlled to assisted MV. A possible example of the AI-driven closed-loop control of sedation and MV is depicted in Figure 3.

Lastly, establishing a “transitioning readiness protocol” that integrates insights from a multidisciplinary team and continuously evaluates both the sedation depth and respiratory mechanics can enable safer and more coordinated transitions, potentially improving outcomes for patients with ARDS.

## 6. Conclusions

Optimizing the transition from controlled to assisted MV in patients with ARDS requires an innovative and tailored approach. Continuous monitoring of the respiratory drive and effort, alongside the sedation levels, is vital to ensure effective spontaneous breathing while minimizing the risks of diaphragm dysfunction and lung injury. Clinicians should adopt a proactive strategy that not only seeks to restore spontaneous breathing but also emphasizes the importance of protecting respiratory muscle integrity and lung function. Embracing advanced monitoring techniques and integrating novel therapeutic options, such as partial neuromuscular blockade, can further enhance patient outcomes. Ultimately, the goal is to achieve a harmonious balance that fosters recovery and rehabilitation in these critically ill patients, paving the way for a more successful weaning process and improved long-term respiratory health.

## Data Availability

Not applicable.
